# Establishing a computational biology flipped classroom

**DOI:** 10.1371/journal.pcbi.1006764

**Published:** 2019-05-23

**Authors:** Phillip Compeau

**Affiliations:** Computational Biology Department, School of Computer Science, Carnegie Mellon University, Pittsburgh, Pennsylvania, United States of America; University of Toronto, CANADA

## Abstract

In a flipped classroom, students complete automated modules to replace a traditional lecture, allowing the time devoted for the lecture to be devoted to constructive tasks reinforcing student knowledge. Yet although the flipped classroom offers a compelling approach for fostering a constructivist, student-centric learning environment, research on the efficacy of flipped classes has been mixed. For that matter, is it possible to successfully flip a classroom in an advanced, heavily specialized course like a bioinformatics algorithms course? Over the past several years, the author has implemented a flipped version of such a course and will discuss some of the successes and pitfalls encountered.

This is a *PLOS Computational Biology* Education paper.

## Introduction: Survivorship bias and the lecture

**Fig 1:**
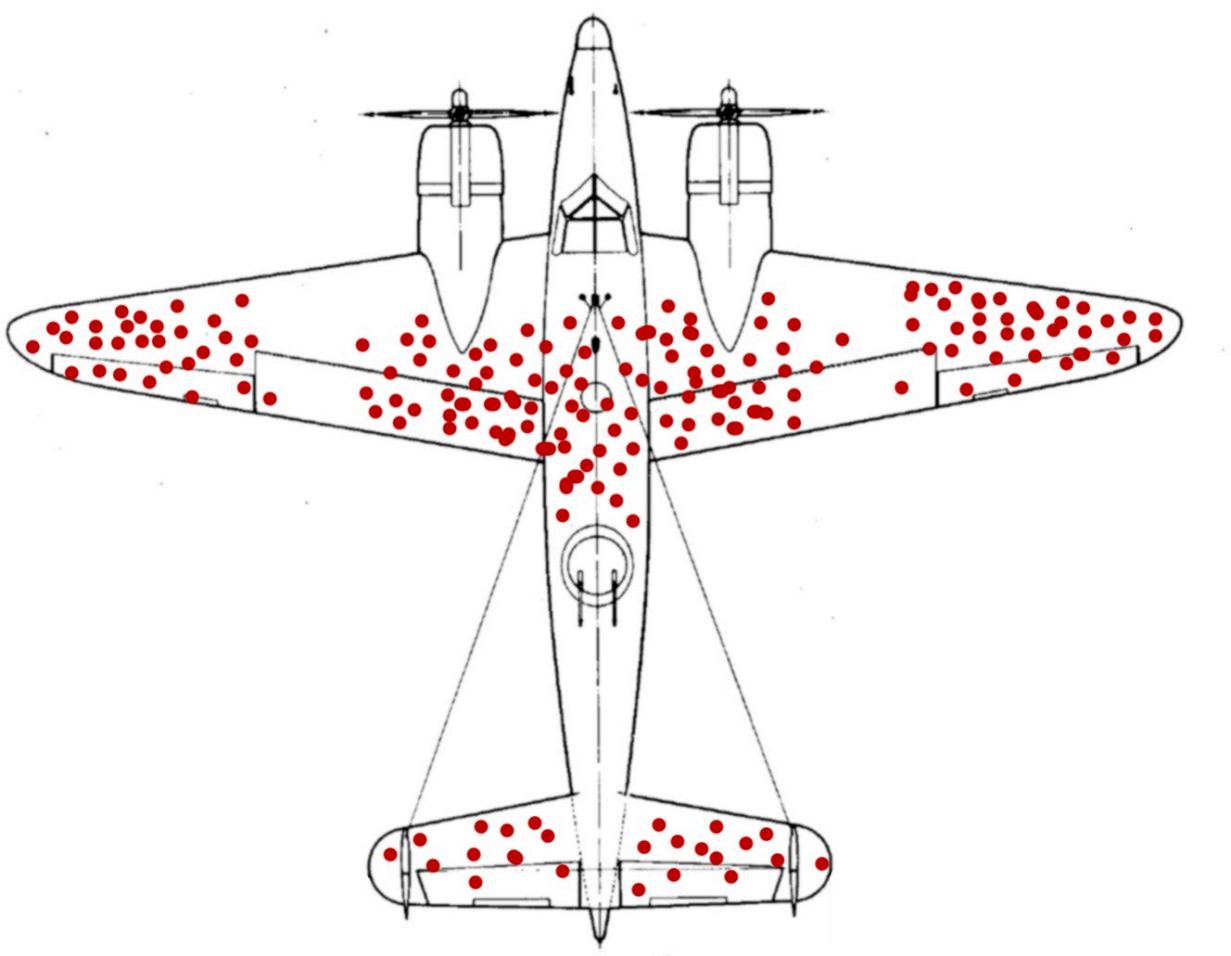
Wald's airplane. A hypothetical diagram of known bullet holes in returning airplanes, illustrated as red points. *Image courtesy*: *McGeddon*, *Wikimedia Commons user*.

Abraham Wald was a Jewish émigré to the United States from Transylvania who was hired by the Statistical Research Group, a Columbia University team that worked in military research during World War II. One problem Wald was tasked with was to examine the bullet distribution of airplanes returning from the battlefield and determine where to strategically place armor, since armoring the entire plane would make it too bulky and consume too much fuel. A hypothetical diagram showing a distribution of Wald's data is shown in Fig 1.

The knee-jerk reaction to Wald's problem is to place armor over the locations of the red dots. Yet Wald's insight was that the armor should go not where the dots are but where the dots aren't. That is, the bullet holes are likely uniformly distributed on the planes, but we don't observe the uniformity in our data because we can't see the planes that were shot down [1].

Wald's missing planes offer an excellent example of “survivorship bias,” in which we are biased toward a particular conclusion because of the invisibility of some subset of our data. The oft-cited ancient example of survivorship bias is from the cynic Diogenes. When shown paintings of shipwreck survivors and asked how he could fail to see divine Providence in their survival, he replied, "Why, I say that their pictures are not here who were cast away, who are by much the greater number."

What does survivorship bias have to do with education? Professors are the survivors of the lecture. We are the "A" students who could sit through an hour-long lecture and internalize the information that our own instructors were bestowing upon us. In fact, we are so enamored with the lecture that we organize our academic conferences around it, lapping up talks for days on end. But have we stopped to ask ourselves whether all this lecturing is best for our students?

The idea that the lecture is an imperfect educational vehicle is neither controversial nor new. In 1984, Benjamin Bloom examined student performance under different classroom conditions. He found that one-on-one tutoring outperformed the conventional 30:1 lecture by approximately two standard deviations [2]. That is, a "C" student in a lecture-based classroom would on average be an "A" student if they had learned the same material via individualized tutoring. I view Bloom's result inversely: there are perfectly capable "A" students that we are transforming into "C" students because we do not have the time and resources to tutor all of them.

Bloom's paper could have rung the death knell of the lecture as the primary mode of teaching in the science, technology, engineering, and mathematics (STEM) classroom. Yet here we are, decades later, still lecturing away and charging more for the experience than ever. What can we do instead?

## What is a flipped classroom?

**Fig 2:**
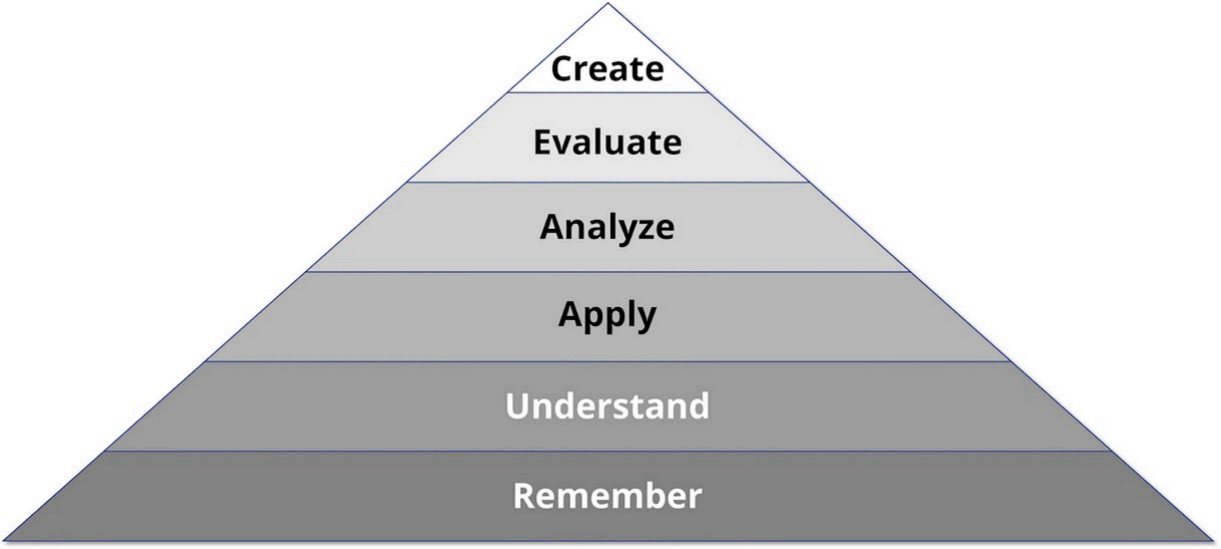
Bloom's taxonomy. A revised 2001 version of Bloom's taxonomy (1956), organized into a pyramid. Higher-level skills proceed upward.

To understand why the lecture might be suboptimal, let us consider another of Bloom's namesake educational psychology paradigms, “Bloom's taxonomy” [3][4], organized into a pyramid shape in Fig 2. We all want our students to move upward in the pyramid, enabling them to apply and evaluate what they have learned within new contexts. Yet where in the typical lecture are we providing them with anything other than—at best—the bottom two levels of the pyramid?

One definition of a “flipped class” is one in which students prepare these bottom two levels on their own, freeing up valuable in-class time for other activities. In the worst case, this time can be used to ensure understanding and provide students with timely feedback on their work. Ideally, class time is devoted to assessments that build higher-level student abilities.

Bishop and Verleger [5] proposed that a classroom is only truly flipped if the materials that students review outside of class are automated. This clarification excludes class structures in which students simply read an article or watch a lecture video without exercises as preclass instruction. Their definition also helps indicate the clear bottleneck in implementing flipped classes. How can we provide automated instruction to students in a still-growing, interdisciplinary STEM field like computational biology?

Fortunately, we are in the midst of a boom in automated online materials. In my own case, I was fortunate enough to codevelop (with Pavel Pevzner) the first massive open online course (MOOC) in computational biology, which first launched in 2013. Yet although I have always been interested in education, we never imagined designing a MOOC. Our dream was to write what we called a "superbook," which we fleshed out into our own acronym: a massive adaptive interactive text (MAIT). This text product, called *Bioinformatics Algorithms* [6], became the engine for our courses. We have described this project elsewhere [7], but we mention it here because a MAIT like *Bioinformatics Algorithms* offers the ideal missing link in implementing the automation component of a flipped class. But now that we have freed up so much class time previously devoted to lecturing, what should we do with it?

## Five years of a flipped classroom for bioinformatics

I have worked on implementing a flipped class using *Bioinformatics Algorithms* as the automated vehicle for the past five years. In what follows, I will discuss the successes and pitfalls that I have encountered along the way (and wish I had known from the beginning). In particular, I profile four specific issues that arose.

I should also make two important notes. First, the account here is predominantly anecdotal, but in my own experience, good teaching is fostered as much from stories of our colleagues on their own experience as it is from research studies. Second, although I profile a course in computational biology, I have tried to identify issues that I believe have broad relevance across disciplines and for students of varying backgrounds.

### Spring 2014: Molecular Sequence Analysis (University of California San Diego)

In spring 2014, I served as a teaching assistant (TA) for this course, with sections for both undergraduate computer science students and PhD bioinformatics students and around 70 students combined. Pavel instructed the course, and we worked together to set up weekly discussions with students.

While completing a weekly interactive reading in *Bioinformatics Algorithms* along with a preclass comprehension quiz, we asked students to draft a write-up of their progress through the text, noting

- every concept that they found challenging;
- every issue that they were unable to resolve on their own; and
- every question (however tangential) that they might have about the material.

We then counted this write-up as part of a required participation grade in the course.

Although the in-class meetings occasionally featured guided group exercises, we largely treated them as open question-and-answer (Q&A) sessions, with the instructor addressing open student questions. After this session, students would be ready to complete a homework assignment building further understanding.

It may seem that running a flipped class would facilitate instructor laziness—just 90 minutes of effort per week! Instead, we found that students asked a plentitude of wonderful questions, many of which we did not anticipate even after thousands of online learners had completed the same material. I have found that it takes the instructor around an hour of time for every 10 students enrolled in a class just to organize their questions before class; in this case, it was a full day's work just to prepare for the in-class Q&A.

Despite our best intentions, the instructor-led Q&A was an awful way to construct a class. The lecture had reared its head: we had returned to an instructor-centric classroom in which 35 students listened to a single instructor spout answers at the front of the room. This setup became particularly troubling in light of the following issue.

**Issue 1: How do we motivate possibly unmotivated students to engage in a flipped class?** In course evaluations, many students were wildly positive, noting that they understood the material far deeper than they could have ever anticipated. A few students despised the course setup. This "bimodality" of student enthusiasm has not been commonly documented in the literature but is an outcome that we have heard from others who have tried to flip a course. Its apparent cause is that the lecture asks so little of its students that growing pains can arise when the flipped classroom starts asking for more. One way of ensuring more universal engagement is to increase peer-to-peer interactions and tie participation to a discussion grade.

### Spring 2016: Fundamentals of Bioinformatics (Carnegie Mellon)

In spring 2016, I was a bright-eyed new teaching-track professor, and I had the opportunity to teach my own version of the course, Fundamentals of Bioinformatics. Six students enrolled in this first offering of the course, which was designed as an elective for first-year master of science (MS) students in our School of Computer Science.

In an effort to facilitate a student-centric classroom, I performed a critical shift in the in-class portion of the course. As the instructor, I posed anonymous student questions and demanded that students must work together to answer the questions while I facilitated the discussion as a "guide on the side" [8].

Despite a small sample size, it was immediately clear from student engagement how much peer responses helped students. Although 90 minutes of peer Q&A did grow monotonous at times, the course felt like an overwhelming success, which was confirmed by superlative course evaluations from all my students.

I felt that my work with developing this course was finished; I was wrong.

### Spring 2017: Fundamentals of Bioinformatics (Carnegie Mellon)

In 2017, Fundamentals of Bioinformatics became a required course for a subset of 30 MS students for whom it would be their primary exposure to computational biology. Building on the success of my previous offering, I split the class into four independent, equally sized groups.

To address the potential monotony of peer Q&A sessions, I restructured the in-class sessions with the following weekly structure:

- A short lecture on a particularly engaging and often tangential topic, often to provide additional context on a fun idea. For example, when covering genome assembly, we discussed how sequencing by synthesis works and how it can be generalized to form paired-end reads.
- A more compact instructor-facilitated peer Q&A, with some group exercises peppered in.
- An instructor and TA–facilitated group discussion guiding students to discover the next week's material. For example, we gave students guided questions probing them to think about how we would compare multiple species using genomic data and leading them to devise computational problems modeling evolutionary tree construction. We then allowed them to brainstorm the algorithms that they could use to construct evolutionary trees. I have been amazed at how well students have performed in these discussions; for example, it is common for a student group to rediscover the classic algorithm UPGMA [9] in just 20 to 30 minutes with limited instructor guidance.

Although we added more structure to the class sessions, teaching students for whom this was a required course and their only contact point with computational biology meant lower student interest. This phenomenon might have been manageable except for the following lesson.

**Issue 2: Selecting the correct cohort size is vital.** Groups of 7–8 students wound up being far too small. Despite restructuring the groups, I tended to have two superstar groups and two groups that struggled to get off the ground. Because of the smaller size, if a group had just a couple of unenthusiastic students, their lack of interest tended to permeate the group. I regret not having spoken to the educational experts at Carnegie Mellon's Eberly Center for Teaching Excellence and Educational Innovation. When I met with them as part of my course postmortem, they immediately informed me that my groups were too small and that the reported ideal cohort size is 20–25 students. (There is unfortunately a paucity of research on cohort size in flipped classes since most such classes opt for a single cohort.)

In retrospect, the issue of cohort size was predictable. Yet the following issue caught me off guard.

**Issue 3: Some students may conceive of a professor as the "font of all wisdom."** Some students may feel that a goal of the traditional classroom is to be wowed by the instructor's knowledge—if the student can only lap an occasional drop from the fountain, then it is the student's problem, not the instructor's. This lesson taught me that the most important part of a flipped course is that it must begin with a sales pitch. To engage all students, the instructor must be willing to spend 30–45 minutes at the start of the course articulating the weaknesses of the lecture, the pedagogical goals of the course, and why the flipped course is being established as a student-centric environment. It must also convince students that exams will test the "higher-level" abilities that are developed in the flipped classroom discussions.

### Spring 2018: Fundamentals of Bioinformatics (Carnegie Mellon)

In the 2018 iteration of Fundamentals of Bioinformatics, I kept the entire class of students in one discussion cohort and lengthened our weekly session to 2 hours. I began each week with a 30-minute review of learning objectives from the week. This measure helped ensure that students were fully prepared for later discussion and that the material was fresh in their minds.

Students then split into small breakout groups of 4–5 students (perhaps the ideal group size for problem solving [10]) and completed three separate instructor and TA–guided sessions:

- common discussion questions from the reading
- challenge problems letting students apply what they've learned to new contexts
- a guided write-up with exercises setting up the next week's material

Note that I returned to employing small breakout groups, but only within the context of a larger cohort. I allowed students to form their own breakout groups, which I was careful to monitor. All groups were engaged and productive, perhaps because each of the three sessions was around 30 minutes, and in part because groups seemed to want to perform well in front of their peers.

An example set of challenge problems encouraged students to apply dynamic programming, a concept that they have learned about only in the context of sequence alignment, to the entirely new problem of virus attenuation based on codon pair bias. What excited me about this series of challenge problems is that all of the breakout groups essentially reached the correct conclusion, and at the end of the discussion, I could tell them that they had rebuilt the engine of a *Science* article with several hundred citations [11]. What more could we want from our teaching lives than to empower our students to replicate scientific research in the classroom?

That having been said, I don't think that Fundamentals of Bioinformatics went perfectly. Why?

**Issue 4: "Course creep" can bloat a course.** I had anticipated that students might become overworked by my lengthening each week's in-class meeting by an hour. Accordingly, I trimmed a week's worth of material from the course and shortened certain homework assignments by a bit. That having been said, 2 hours on one topic can run the risk of exhausting already strung-out students; I would prefer a structure with two 60-minute sessions. If we start asking our students to learn what we are teaching them at a deeper level, we may simply have to sacrifice some breadth.

## Conclusion

**Fig 3:**
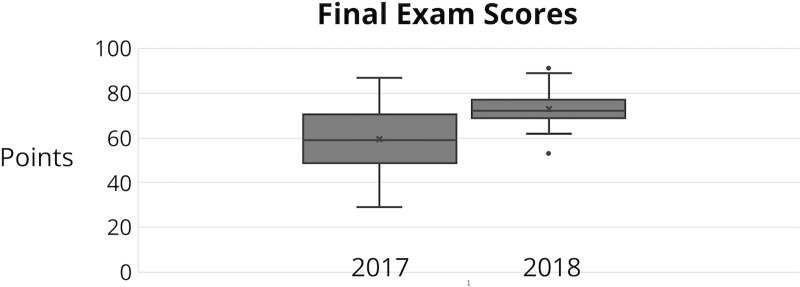
Comparison of student performance with larger cohort size and breakout groups. Comparing final exam scores for Fundamentals of Bioinformatics in 2017 and 2018 reveals a significant upward trend in scores, especially for the bottom 75% of learners. Both exams were comprehensive, of comparable difficulty, and graded on similar rubrics out of 100 points possible.

This was all a lot of work to devote to flipping a single course, but I was encouraged to see how my final exam scores in 2018 compared with those of 2017 (Fig 3.). The two exams were of comparable difficulty with similar rubrics, yet there was a stark increase in student performance. I attribute this uptick to better structured in-class sessions, as well as a better sales pitch on why students should trust in the flipped class and how their participation would correlate directly to their exam performance. I would note that my final exam is designed to be quite challenging and test students' ability to apply what they've learned to problems in new contexts—I cannot imagine seeing the results from 2018 with a traditional lecture.

Although I feel that my course has been a success, research results on flipped courses tend to be mixed [5]. Why might this be the case?

Despite huge efforts into online education, existing materials for automating students' preclass learning can be spotty. Asking every instructor to construct their own materials is perhaps unrealistic.

The paucity of online course materials for many fields is evident, but it is easy to underestimate the importance of designing the in-class sessions. It took me a few runs of a flipped classroom to realize that it may be better to design a flipped classroom by implementing "backward design," [12] in which we first establish learning objectives to help us design the in-class sessions and only then identify what online materials we can use to flip the classroom.

Furthermore, the lecture is stable in that it can proceed from point A to point B with few surprises. But a mistake in establishing a flipped class (such as my mistake of setting the cohort size too small) can magnify quite quickly.

When implemented well, a flipped class is a rewarding experience, but there are many ways to build student-centric classrooms. In “active learning,” we take a constructivist approach to student learning, finding opportunities for students to learn material by doing whenever possible. This is the "just-in-time" premise of *Bioinformatics Algorithms*, as well as the guided discussion and challenge problem sessions that I give students in Fundamentals of Bioinformatics. In fact, Freeman and colleagues [13] surveyed over 200 studies and found that students in courses with active learning outperform those in traditional lectures by around half of a standard deviation, with a 50% reduction in failure/withdrawal rates as well.

In fact, Jensen and colleagues have argued [14] that what makes a flipped classroom typically outperform a lecture may be attributable to its incorporation of active learning and that active learning can be integrated into a lecture format with similar outcomes to the flipped classroom. This result gives hope to instructors who may recognize the pitfalls of the traditional lecture but who have difficulties finding resources or time to flip their classrooms overnight. (I count myself as one of these instructors, having 3 years of experience in adding active learning to a lecture-based programming course.) In this light, active learning may also represent an intermediate testing ground with less investment between a traditional lecture and the flipped classroom.

Regardless of the teaching paradigms we choose, I hope that we can find ways to end the traditional lecture as it currently exists in most institutions. Education might be one of the last fields of human endeavor to be disrupted by automation, but it will come. As instructors, we have a rare opportunity to either leverage this automation or find ourselves cast to the scrap heap.
